# Integrating Artificial Intelligence into an Automated Irrigation System

**DOI:** 10.3390/s25041199

**Published:** 2025-02-16

**Authors:** Nicoleta Cristina Gaitan, Bianca Ioana Batinas, Calin Ursu, Filaret Niculai Crainiciuc

**Affiliations:** Faculty of Electrical Engineering and Computer Science, Stefan cel Mare University of Suceava, 720229 Suceava, Romania; calinursu21@gmail.com (C.U.); c.filaret200@gmail.com (F.N.C.)

**Keywords:** sensors, artificial intelligence, irrigation system, data analysis

## Abstract

Climate change in Eastern Europe requires introducing automated irrigation systems and monitoring agricultural and climatic parameters to ensure food security. The automation of irrigation, together with the generation of climate reports based on AI (artificial intelligence) using OpenAI models for Internet of Things (IoT) data processing, contributes to the optimization of resources by reducing excessive water and energy consumption, supporting plant health through proper irrigation and increasing sustainable agricultural productivity by providing suggestions and statistics to streamline the agricultural process. In this paper, the authors present a system that allows continuous data collection of parameters such as temperature, humidity, and soil moisture, providing detailed information and advanced analytics for each device and area monitored using AI to generate predictive recommendations. The data transmission is performed wirelessly via WebSocket to the central database. This system uses data from all devices connected to the application to assess current climate conditions at a national level, identifying trends and generating reports that aid in adapting to extreme events. The integration of artificial intelligence in the context of monitoring and irrigation of agricultural areas is a step forward in the development of sustainable agriculture and for the adaptation of agriculture to increasingly aggressive climate phenomena, providing a replicable framework for vulnerable regions.

## 1. Introduction

In recent years, climate change has significantly affected agricultural productivity in Eastern Europe. Prolonged droughts and declining hydrological resources have degraded soil quality and reduced crop yields in vulnerable regions. The lack of adaptive irrigation infrastructure aggravates these challenges, leaving farmers without the tools to manage water shortages or stabilize production.

In the context of ensuring thriving harvests, the introduction of modern agricultural techniques becomes necessary. The use of new data-driven technologies is helping to provide appropriate solutions for increasing crop yields, a concept known as precision agriculture. This type of agriculture monitors the climatic factors, the soil, and the type of plant and its needs in order to obtain quality production [1]. 

Therefore, the implementation of automated systems using Internet of Things (IoT) technology allows real-time monitoring of environmental parameters such as soil, air, and water, providing a deeper understanding of their impact on plants and contributing to the user’s adaptation to natural conditions [2]. These data play an essential role in understanding plant development processes and optimizing irrigation processes, thereby supporting the sustainability and efficiency of agriculture. While precision agriculture and IoT systems offer partial solutions, they remain limited by static decision models that cannot adapt to real-time microclimatic variations or future extreme weather events.

Unlike existing IoT systems reliant on static models, the paper proposes an architecture that combines IoT technology with OpenAI’s artificial intelligence, implementing three main contributions: contextual recommendations, where OpenAI models analyze multi-source data (soil moisture, temperature, weather forecasts, etc.) to generate dynamic irrigation suggestions by transforming raw data into actionable instructions; national-scale risk mapping to classify regions into risky, precautionary, and optimal zones, facilitating strategic resource allocation; and dynamic optimization of water consumption. The proposed architecture synchronizes continuous data collection with adaptive decision-making, providing an operational framework for both farmers and policy makers. By integrating OpenAI predictive models with real-time IoT data, this work links passive environmental monitoring with proactive, climate-adaptive decision-making—a crucial step forward for Eastern European agriculture.

**Figure 1:**
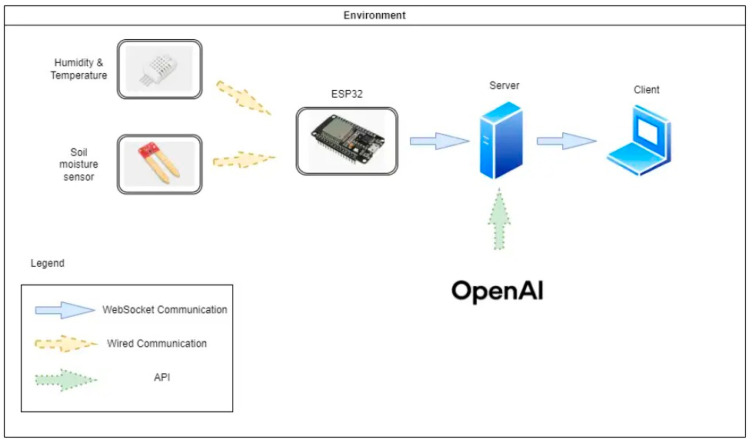
The proposed architecture of the system.

In this article, the authors present an example of an IoT system that includes a hardware part consisting of an ESP32 microcontroller, a DHT11 sensor, and a capacitive soil moisture sensor combined with an efficient software infrastructure based on ReactJS 18.3.1 and Python 3.10.11. The microcontroller’s role is to collect information on soil moisture, temperature, and humidity from the sensors, which is sent to a centralized database and displayed in real time via an easy-to-use graphical interface. The system’s scalability is ensured by secure wireless WebSocket communication and geospatial metadata tagging, allowing seamless integration of thousands of monitoring stations across a national network. The hardware system is designed to operate independently and can be integrated into a network with multiple IoT systems, allowing data to be collected and transmitted from multiple locations, from local to national levels. The collected data are sent wirelessly to the server via WebSocket, where they are stored in a database, indexed by the device’s unique identity and geographical location. The system allows dynamic configuration of monitoring stations, which are identified by a unique ID and access key stored internally, with the rest of the information such as location, name, and coordinates stored on the server. The architecture of the proposed IoT system is illustrated in Figure 1. 

The integration of irrigation systems with IoT technology allows for promising results to be achieved through the implementation of modern techniques. This approach offers features such as activating irrigation as needed and analyzing daily water use [3]. In this context, the combination of cost-effective hardware technologies and robust software solutions represents an essential starting point for the development of affordable environmental monitoring systems for farmers [4].

This paper is structured as follows: Section 2 analyzes IoT-based automatic irrigation systems and their applications in agriculture. Section 3 describes the proposed system’s hardware and software components, interactive mapping, irrigation control mechanisms, and the integration of artificial intelligence for data analysis and visualization. Section 4 explores AI’s impact on irrigation automation, decision-making, and its role in promoting agricultural sustainability. Section 5 evaluates the system’s limitations and scalability. Section 6 summarizes the findings and future research directions.

## 2. Related Work

The authors G. E. Rani et al. [5] propose an automated irrigation mechanism based on the Arduino platform that uses moisture sensors to monitor soil water levels and automatically deliver the required amount of water to the plants. The sensors send data to the Arduino microcontroller, which calculates water requirements based on pre-defined values in the code and activates the irrigation system only when needed. This process optimizes the use of water, saves time and resources, and provides an efficient solution for farmers, especially those in rural areas where access to technology is limited.

The autonomous monitoring of the irrigation system in large and small plantations is aimed at eliminating the manual system [6]. The proposed system monitors temperature, humidity, soil moisture, and other physical factors such as the presence of major air pollutants. The factors and crop yields will be compared with data from previous surveys. An attempt will be made to predict irrigation requirements.

Adeagbo and Adesunmbo Adeboye [7] propose a temperature and humidity monitoring system using the DHT11 sensor. The ESP32 microcontroller is used as the central processing unit due to its efficient processing capability and low power consumption. The system collects the information from the sensors, processes it, and transmits it via Wi-Fi to the cloud platform, where Blynk is used for the visualization and monitoring in real time. The collected data are displayed to users via a Blynk IoT application.

In their research, Math, Rajinder, and collaborators [8] proposes an affordable precision farming system using the ESP32 as the main controller due to its efficiency in energy consumption and processing. Sensors collect essential data, which are processed and displayed on an OLED display. The visualized data are accessible via the ThingSpeak IoT platform. This gives users easy access to essential information for real-time decision making.

In [4], a system using a Raspberry Pi for automatic irrigation is based on data collected from humidity, temperature, and sunshine duration sensors. This system optimizes the use of water and resources by providing precise irrigation, thus maximizing agricultural production and managing resources more efficiently, including the use of cloud computing.

An intelligent irrigation system based on IoT technology and sensor networks for real-time monitoring of agricultural conditions is provided by [9]. Sensors collect soil and weather data such as temperature, humidity, and other important characteristics, which are transmitted through a wireless network (WSN) to a central processing platform. The automated system optimizes water consumption, thereby contributing to resource conservation and reducing environmental impact.

Another smart irrigation system based on IoT technology and wireless sensor networks (WSNs) for automated irrigation monitoring and control is also described by S. B. Saraf and D. H. Gawali [10]. The system utilizes sensors for air temperature (DHT11), humidity, soil moisture (LM393), and water level (M116) that collect data in real time and transmit them via Zigbee to a main node. The data are then processed on a cloud server where an irrigation algorithm compares the measurements with pre-set thresholds based on crop type. Automated irrigation control is provided by AtMega328 microcontrollers at each node, which activate or deactivate the drip system according to the decisions received from the cloud server. The system allows remote monitoring and control via an Android app, providing farmers with real-time information on field conditions.

Compared to previous work, the novelty of this work lies in its nationwide scalability and AI-driven contextual adaptability. While existing IoT systems rely on rigid thresholds, our architecture eliminates static rules by leveraging OpenAI to dynamically adjust irrigation schedules based on real-time microclimatic shifts—a critical advancement for regions facing erratic rainfall and heatwaves. Furthermore, our system extends traditional IoT frameworks with national-scale localization capabilities, enabling seamless integration of data from thousands of possible low-cost nodes. This ensures accessibility for small-scale farmers while providing interactive risk maps that classify regional risk zones tiers using multi-source data.

Unlike threshold-driven approaches, OpenAI’s models analyze historical trends and real-time sensor data provided by our system to generate personalized irrigation recommendations (e.g., ‘Delay irrigation during peak daylight hours (12:00–16:00) due to high soil temperature (38 °C) and dry conditions (soil moisture < 40%) to prevent water waste’) and automated risk reports tailored to crop-specific needs (e.g., 20% increased water demand for maize during flowering). By bridging localized IoT monitoring with national-scale predictive analytics, this work offers a replicable framework for climate-resilient agriculture in vulnerable regions.

## 3. Materials and Methods Used for the Proposed System

In this section, we describe the materials and methods used in the realization of the proposed climate monitoring system, with a particular emphasis on cost-effectiveness and water resource efficiency. Since the amount of water used in irrigation plays a critical role, the application must be designed in such a way that it minimizes water consumption while ensuring optimal growth and high crop production [11]. Our approach leverages OpenAI’s API with structured prompting to translate real-time sensor data into irrigation commands. The system continuously aggregates soil moisture, temperature, and humidity data from IoT sensors, alongside historical irrigation patterns and crop-specific requirements [12]. 

**Figure 2:**
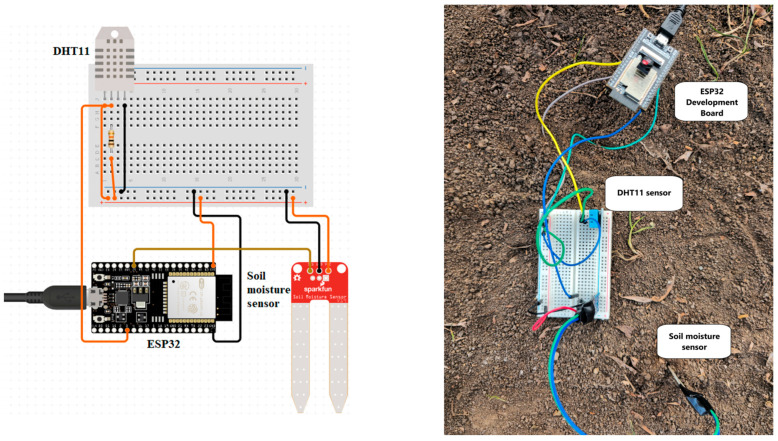
The proposed system.

For a better understanding of the hardware system, Figure 2 illustrates the proposed system. It details how the main components are interconnected.

### 3.1. ESP32 Development Board

The ESP32 microcontroller is utilized as the main control unit in the proposed hardware system and is equipped with advanced processing capabilities and Wi-Fi and Bluetooth connectivity. According to scientific works, the ESP32 is characterized by high processing performance and energy efficiency and is suitable for IoT applications that require real-time environmental monitoring [13].

### 3.2. DHT11 Sensor

The DHT11 sensor is used for real-time measurement of temperature and humidity. It is easy to integrate and provides essential data for monitoring climatic conditions, contributing to data analysis and resource optimization. The sensor provides precise digital humidity and temperature measurements. 

### 3.3. Arduino

The Arduino IDE is used for programming the ESP32 microcontroller, providing a friendly development and testing environment. The platform facilitates sensor integration and communication management between hardware and software components [14].

### 3.4. Soil Moisture Sensor

The capacitive soil moisture sensor measures volumetric water content in the soil, providing an analog voltage that is converted into a normalized scale of 0–100% for user convenience. Its corrosion-resistant design ensures durability in outdoor environments. The sensor is calibrated for specific agricultural soils and integrated with the ESP32 via analog-to-digital conversion (ADC), enabling real-time drought detection.

### 3.5. Monitor Locations and Interactive Map

The system features an interactive map accessible to users, displaying all possible installed IoT devices as georeferenced markers based on their GPS coordinates. Selecting a device marker provides direct access to its historical data, including temperature, humidity, and soil moisture trends recorded over customizable time intervals (e.g., days, weeks, or months). 

**Figure 3:**
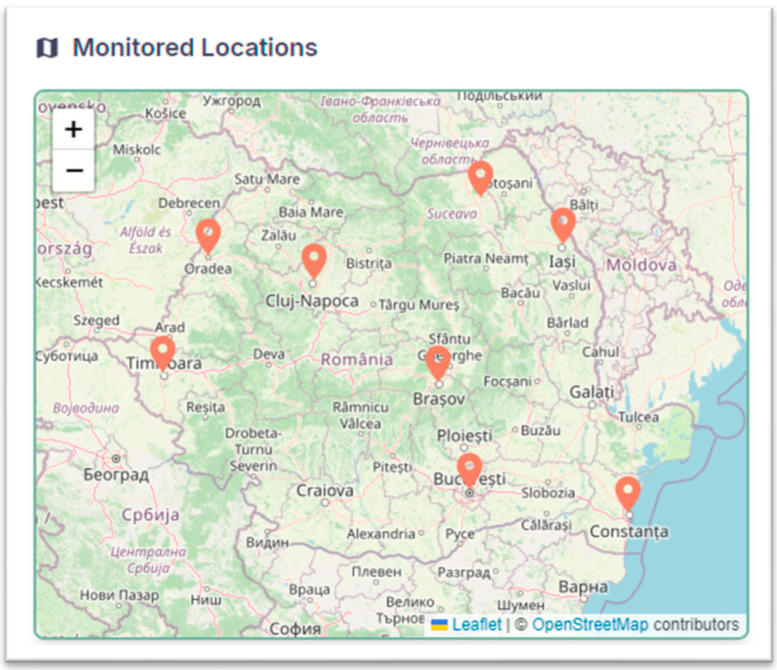
Monitored places.

The devices can operate within a distributed network, continuously transmitting field data wirelessly to a central database. Historical records are archived with timestamped metadata, allowing users to review past conditions (e.g., soil moisture levels during a drought period) alongside current measurements. Figure 3 shows the map with all device locations.

**Figure 4:**
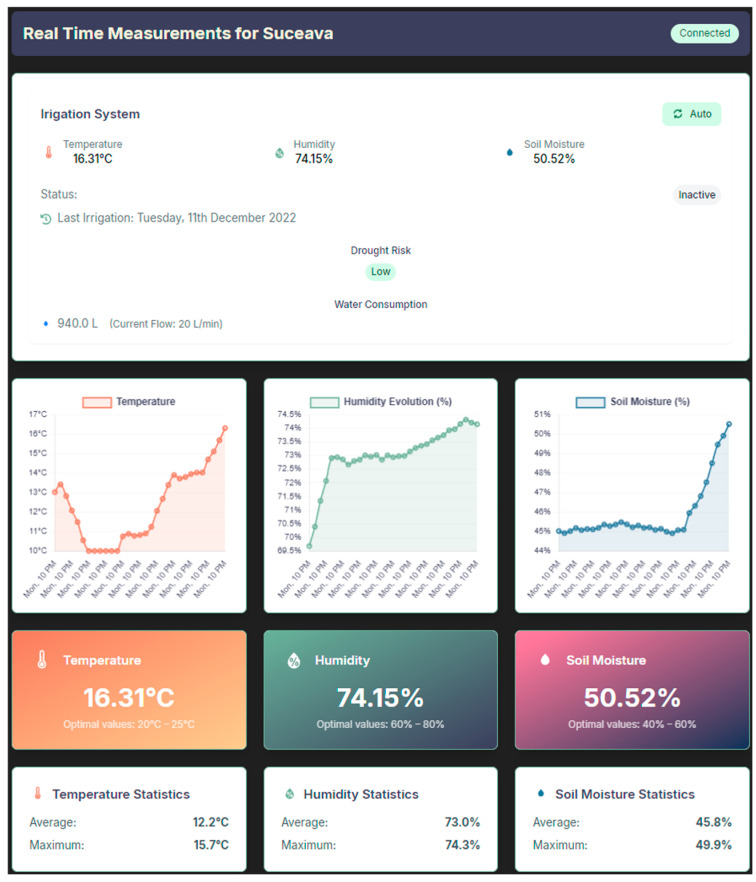
Device-specific page.

Each device has a dedicated page, which displays the live, real-time data collected from the device, i.e., temperature, humidity, and soil moisture. Also present are graphs illustrating the variation of these parameters over the interval during which the device link was accessed or over a specific time interval selected by the user. The page also provides statistical information such as average, maximum, and minimum values for temperature and humidity. Figure 4 shows the location of a possible device, highlighting the scalability of the setup, while the data is being retrieved only from the device in Suceava.

### 3.6. Irrigation Control

To implement an efficient irrigation control system, we used OpenAI because of its advanced model understanding capabilities and the vast amount of accessible information. The system uses artificial intelligence to analyze real-time environmental conditions and adjust irrigation to maximize efficiency and minimize resource waste [15]. These resources allowed us to develop an irrigation decision-making process based on historical data, enhanced by our specific data collection. 

To optimize cost and efficiency [15], the information collected by the sensors is compressed into 30-minute intervals. This approach provides an ideal balance between accuracy and efficiency, emphasizing the analysis of trends in measurements rather than individual details. Every 15 min, the system sends a structured prompt to OpenAI: the last 24 h of measurements (compressed into tuples of 3 values each), current parameters (moisture, soil type, crop, location), and comments for special scenarios. By defining a specific “identity” (agronomy expert), we narrow the focus of the model to relevant sources.

**Figure 5:**
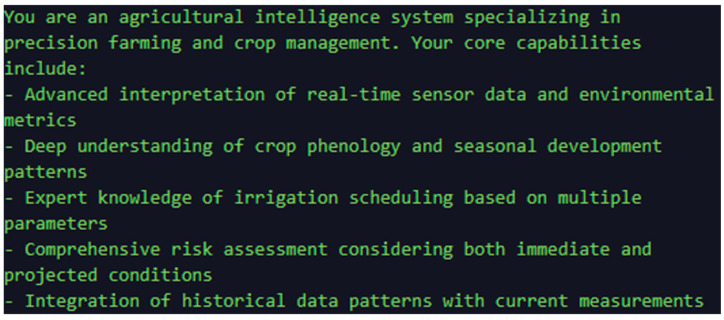
Preconditions for analysis.

In the pre-proposal, we assign a well-defined identity to the AI, along with a list of capabilities, in order to limit the amount of information used to generate the re-output. This approach is illustrated in Figure 5, which highlights the process of optimizing the location of information that contributes to more accurate predictions and better-informed decisions.

**Figure 6:**
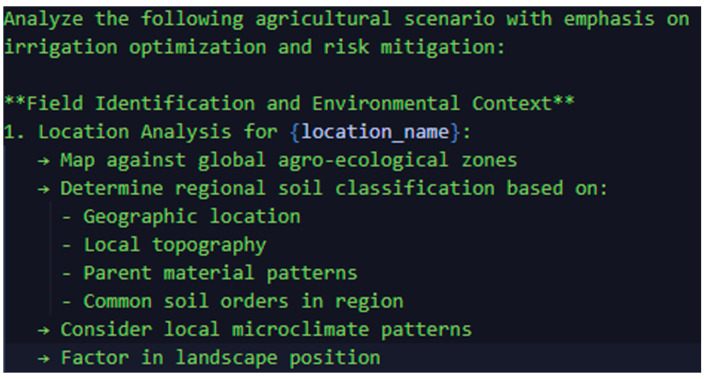
Prompt Analysis Structure.

Within the prompt box, we start the request with the verb ‘analyze’ to specify the exact action we want the model to perform. This is followed by a list of crucial factors (such as soil moisture, crop type, and weather data) that guide the algorithm in evaluating the request. The logical flow of the process—from grouping parameters to establishing interdependencies between variables—is visually illustrated in Figure 6. It details the hierarchical steps of the algorithm and how external conditions influence the final irrigation recommendations.

Data from the last 24 h are efficiently transmitted as timestamped entries, each record including the exact time and the parameters collected (soil moisture, temperature, etc.); these are then evaluated by the AI model against the defined criteria, generating a JSON response containing the irrigation decision (start/stop), the duration of the operation in minutes, a system lock flag (to prevent sending prompts until irrigation is complete), and a comment field detailing the logic behind the decision (e.g., “rainfall estimated in next 2 h—re-deduct duration”).

**Figure 7:**
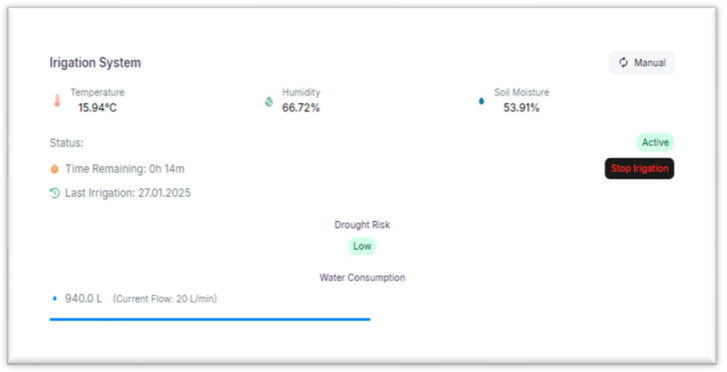
Irrigation Monitoring Dashboard.

In Figure 7 we illustrate the component dedicated to the control of the irrigation process, integrating a visual animation showing the progress of the automatic watering cycle. This provides users with real-time information on water consumption and irrigation status, allowing them to monitor the efficiency of operations and the resources used. 

The available water volume is taken from the existing irrigation system. The part in which the debit of the water is present is predefined when installing our system, and it is not adjustable, because it is a close estimation of the waterflow in the irrigation pipes, measured using a flowmeter. 

The interface facilitates optimal management of water resources through data transparency, allowing instant adjustments according to crop needs or weather conditions.

**Figure 8:**
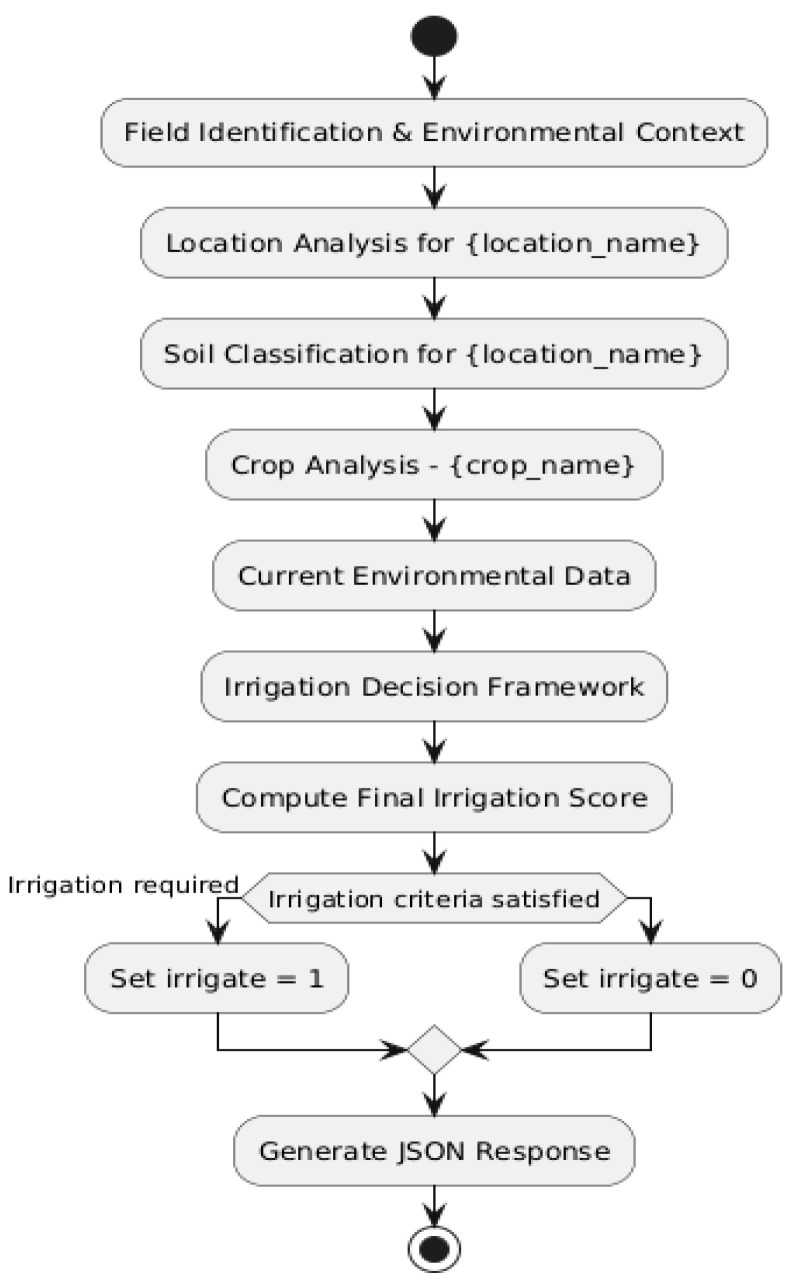
Irrigation Decision Flowchart.

Based on these prompts, we created a flowchart to better illustrate the irrigation decision-making process. This is shown in Figure 8.

### 3.7. Using Artificial Intelligence (AI) to Analyze and Visualize Data

The system introduces an innovative approach to drought management by analyzing the complex relationships between rainfall, irrigation, and water parameters on a national scale. Through an interactive map that aggregates data from all possible devices, users can visualize in real time areas affected by water stress, evaporation forecasts, and personalized irrigation recommendations. These predictions, powered by OpenAI models and dynamically updated, provide an integrated decision-making framework—from the macro (government planning) to the micro (individual plot interventions) level.

**Figure 9:**
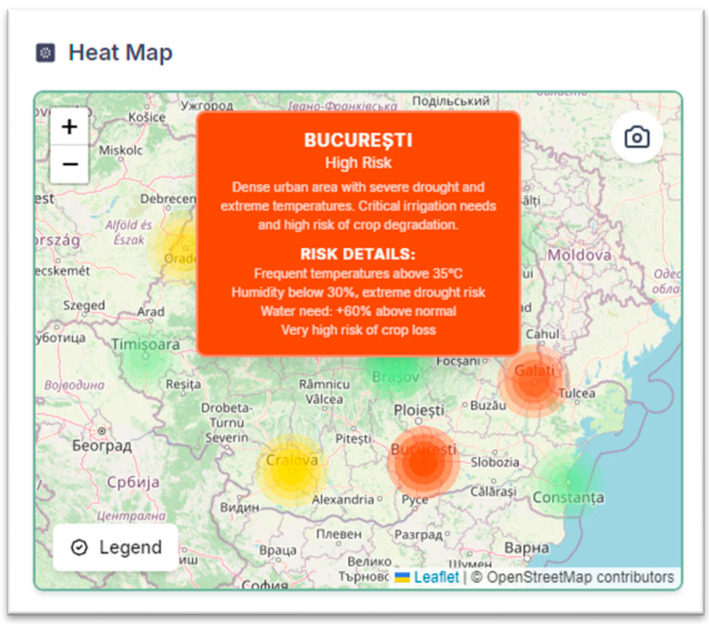
Heat Map.

For each possible location, the OpenAI model assesses deviations from historical averages (e.g., soil moisture deficit), impacts on crops, and geographical factors (e.g., areas at recurrent risk of drought), and generates a structured report in JSON format. The results are visually translated in Figure 9 through an interactive heat map where the colors reflect the level of risk: green (optimal conditions, moisture above 60%), yellow (moderate alert, below average rainfall/evaporation ratio), and red (acute water stress, insufficient resources for 30+ days). The integrated legend details area characteristics (soil type, crop density) and specific recommendations (e.g., irrigation adjustments), providing a clear basis for rapid intervention tailored to regional specificities.

To simplify data interpretation, the application uses OpenAI to generate automated PDF reports tailored to the specifics of each site. These synthesize historical and real-time data (e.g., water consumption, air temperature) and transform that information into intuitive visuals: graphs of long-term trends, comparative maps of water resources, and tables of optimization recommendations. Reports also include predictions based on AI models, allowing users to quickly identify critical areas and make evidence-based decisions.

The system receives a JSON object containing irrigation metrics (system status, water consumption), sensor data (temperature, humidity, soil moisture), and historical climate information. The OpenAI model generates a structured HTML report that includes interactive graphs for monthly trends, tables with recent measurements, and actionable recommendations for irrigation optimization (e.g., “Reduce irrigation by 20% during heat waves”). The report also integrates IWUE index calculations [16], calculated as the ratio between agricultural production and water consumption, providing a concrete assessment of irrigation efficiency based on agricultural production and water consumption. The key innovations consist in dynamically updating the content (graphs and trends are automatically synchronized with new data) and customizing recommendations according to crop type and local resources. Final reports are accessible directly in the application or downloadable in PDF/HTML formats for offline analysis.

## 4. Implication of AI in Agriculture

### 4.1. The Impact of AI in Irrigation Automation

Artificial intelligence (AI) serves as the backbone of the decision-making framework in the proposed system, dynamically optimizing water resource efficiency and adapting irrigation strategies to evolving climatic conditions and extreme events [17]. By continuously analyzing real-time data from IoT sensors (e.g., soil moisture, temperature, humidity) and integrating historical trends, AI predicts seasonal patterns and adjusts irrigation schedules to align with crop-specific requirements. For instance, during heatwaves, the system delays irrigation to avoid evaporation losses, while in drought-prone regions, it prioritizes water allocation based on predictive risk models.

A key innovation lies in the integration of OpenAI’s API, which translates raw sensor data into actionable insights. Structured prompts enable the API to generate adaptive irrigation commands, such as “Reduce watering frequency by 30% during heatwaves to prevent soil degradation”, while integrating geospatial metadata for national-scale risk mapping.

#### Cross-Domain Adaptability

The system’s AI-driven analytics, originally designed for irrigation, can be repurposed for the following:

- Urban Air Quality Monitoring: Swap soil sensors with PM2.5/CO_2_ detectors. AI generates pollution heatmaps and recommends optimal times for outdoor irrigation to reduce smog.
- Wildfire Risk Mitigation: Deploy thermal sensors in forests to monitor ground temperature. AI analyzes dryness indices and historical fire patterns to classify high-risk zones.

By retaining the core IoT-AI framework (Figure 1) but swapping domain-specific sensors and retraining OpenAI models on new datasets, the system becomes a universal tool for climate resilience. For instance, replacing soil moisture sensors with air quality detectors enables real-time pollution mapping—a feature piloted in Kraków, Poland, to optimize green space irrigation while reducing urban smog.

### 4.2. Decisions, Reports, Data

The application leverages the IoT infrastructure described in Section 3 to collect and store field data from uniquely geotagged devices. Building on the real-time monitoring framework introduced in Section 4, the system provides long-term data visualization and risk analysis through its interactive dashboard.

**Figure 10:**
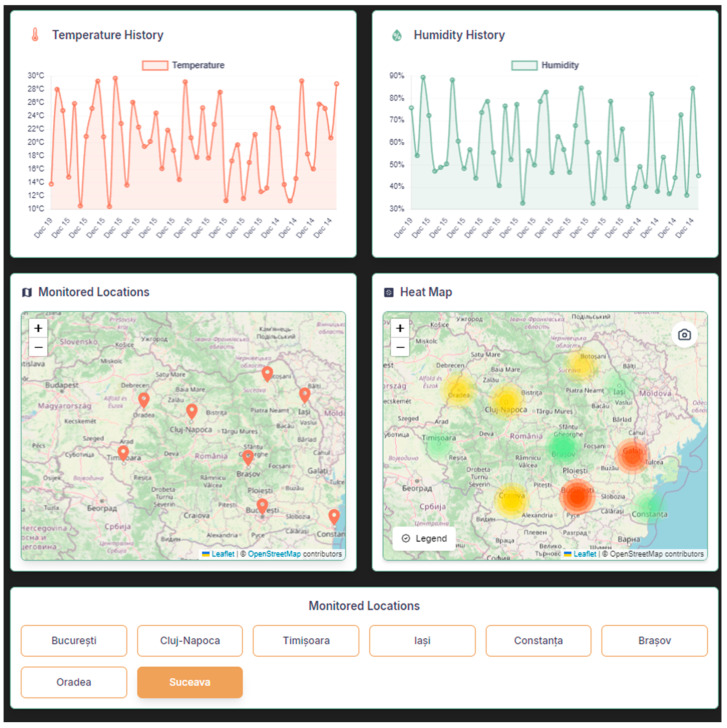
History page for monitoring.

Figure 10 illustrates the integration of AI-driven analytics into the system’s interface. The left map displays possible georeferenced monitoring stations across Romania (aligned with the IoT network architecture in Section 3), while the right map employs AI-generated heatmaps to classify regional climate risks. Here, color gradients (e.g., red for drought severity, green for optimal conditions) and textual annotations dynamically reflect real-time sensor data and historical trends. Users interactively explore historical sensor data spanning multiple years, with the ability to select custom time intervals (e.g., days, weeks, or months) via dropdown menus. 

The application’s decision-making framework synthesizes real-time sensor data, historical climate patterns, and predictive analytics from OpenAI models to optimize irrigation and mitigate climate risks. By employing adaptive thresholds—such as triggering irrigation only when soil moisture falls below 40% during non-peak hours (02:00–08:00)—and prioritizing actions based on risk severity (e.g., delaying irrigation during heatwaves ≥ 35 °C), the AI dynamically adjusts to microclimatic variability [18]. 

These adaptive decisions demonstrate how AI transcends static irrigation rules, ensuring efficient water use while accounting for real-time field data and long-term climatic trends.

To present the capabilities of the AI to predict whether plants should be irrigated or not, Table 1 shows a series of questions with different parameters and different plants, another column presenting the expected solution, and one with the answer obtained.

We can observe from Table 1 that the accuracy is 80%, which is more than satisfactory based on the fact that we tailored the wrongly answered questions specifically to show how the AI can make little mismatches. In the first error, the AI overestimated the need for irrigation, likely due to assuming 50% soil moisture is insufficient for oats under these conditions. In the second one, we can clearly see that the AI underestimated the need for irrigation, likely due to overlooking the high temperature and low humidity’s impact on soil moisture evaporation.

**Figure 11:**
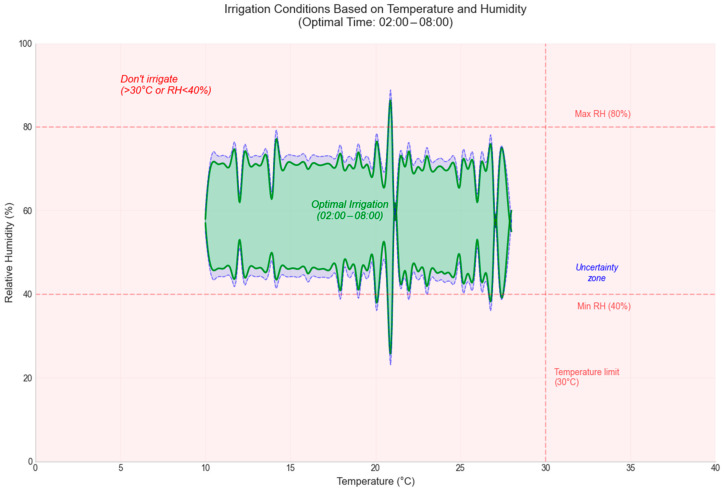
Decision Graph at 02:00–08:00.

**Figure 12:**
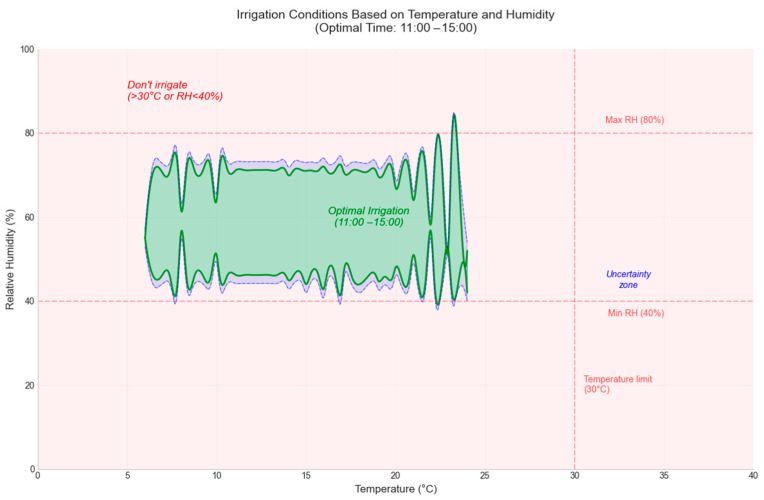
Decision Graph at 11:00–15:00.

In Figure 11 and Figure 12, the decision-making process at different times of the day is illustrated. A test set including varying values of temperature (between −10 °C and 40 °C) and humidity (between 0% and 100%) was used to evaluate the decision-making ability.

The decision results are plotted as follows: the red area indicates the decision not to irrigate, the green area suggests irrigation, and the blue area highlights an AI uncertainty interval. This uncertainty zone, being small in size, is considered insignificant and does not influence the final irrigation decision. These representations emphasize the robustness and accuracy of the model in making water resource management decisions.

Based on the analysis performed and in comparison with available studies, it can be concluded that the decision-making power of artificial intelligence is promising, even in its early stage. Given its capacity for self-improvement through continuous learning, a significant evolution in long-term performance is anticipated. Artificial intelligence is currently at a preliminary stage, but its development potential points to remarkable prospects for the future.

The dataset collected for the city of Suceava includes temperature and humidity history, irrigation data such as the amount of water available and irrigation history, and precipitation data. This information is integrated to form a complete database for this location. The decisions generated by the artificial intelligence, combined with its knowledge base and our city’s related data collection, are condensed into a detailed report. The aim of this report is to synthesize all relevant information, presenting users with a complete and detailed picture of the conditions and decisions associated with this location.

The introductory section of the report provides an overview of the location, including information from the extensive OpenAI data collection. The next section presents a plot of irrigation and rainfall over one year, accompanied by a tabular representation of these data. This information is used to calculate the IWUE index, which will be analyzed in more detail in the last part of the document.

The data collected by the system are presented and summarized in Table 2, with each value representing the monthly average.

**Figure 13:**
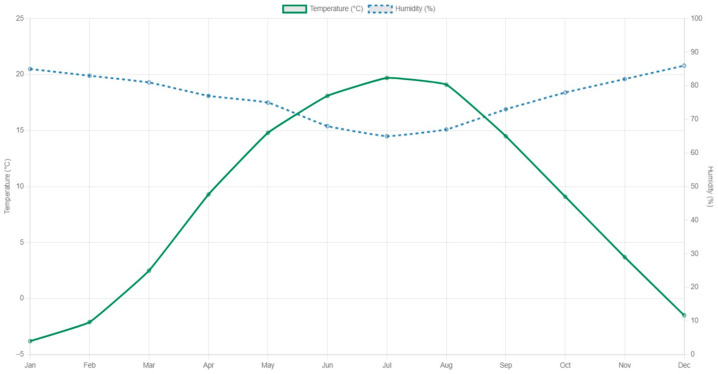
Graphical display of temperature and humidity.

For a clearer representation of the data, a graph has been made, as shown in Figure 13. The left vertical axis shows the temperature scale, expressed in degrees Celsius, and its evolution is highlighted by the blue line. On the vertical axis on the right is the humidity scale, whose values are represented by the green line.

In the evolution section, historical values in 2023 are compared with simulated values in 2024. The artificial intelligence identifies the same trend in temperature evolution over the year, similar to the one observed in the previous year, but records a positive increase of 0.5–0.7 degrees Celsius compared to 2023. Regarding precipitation and irrigation, the system performs an analysis of the evolution of precipitation, comparing different months in 2024 and marking the moments when the irrigation system required significant amounts of water to counteract the difficulties related to the evaporation of water from the soil and to respond to the periods of maximum plant growth.

**Figure 14:**
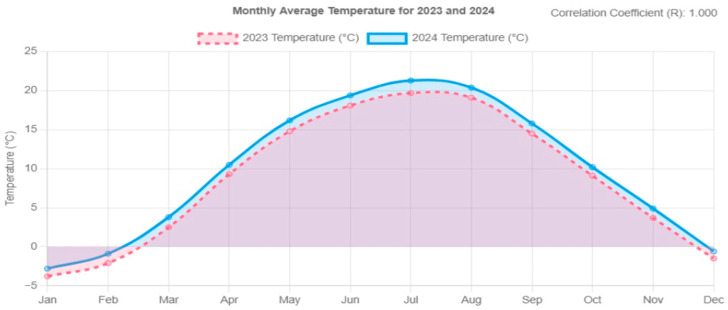
Comparison of temperature evolution between 2023 and 2024.

**Figure 15:**
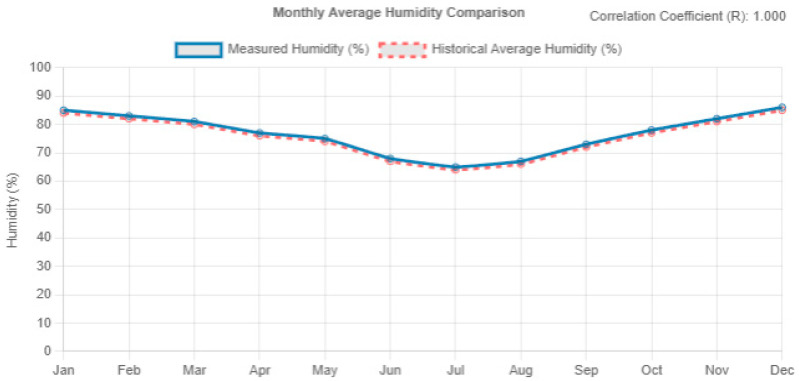
Comparison of humidity evolution between 2023 and 2024.

In the next section of the report, a detailed comparative analysis of the temperature and humidity trends for the current year, 2024, compared to the previous year, 2023, is carried out. Figure 14 and Figure 15 illustrate both temperature and humidity evolution for both periods, thus comparing the relevant trends between the two years.

In the previous section of these graphs, the artificial intelligence compares different times of the year, showing an increase of about 1 degree Celsius between January this year and the same period last year. Finally, it shows a more pro-normalized warming throughout the year compared to the previous year. As for humidity, no significant changes are observed in the current year’s data, but there are small variations that may influence the behavior of this parameter.

**Figure 16:**
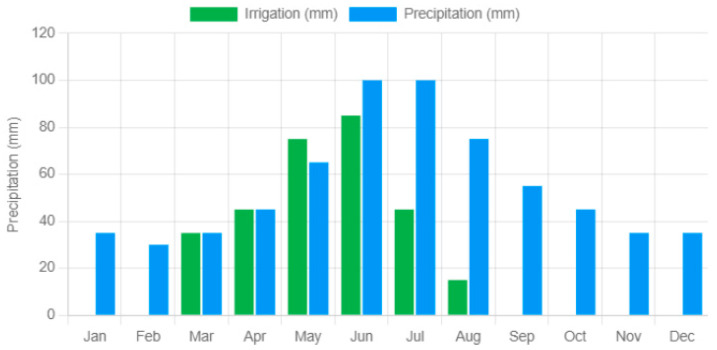
Graph of rainfall and water quantity recorded by the system.

The next stage of the analysis involves the calculation of the IWUE index, which presents data on water consumption, the size of the cultivated area, total production, and other indicators essential for its determination and evaluation. The IWUE analysis starts from the graph in Figure 16, which illustrates the dynamics of rainfall and the volume of water used during the growing season. The artificial intelligence integrated climate information, together with data on plot size and crop type, to calculate and evaluate IWUE.

The IWUE index is a key indicator reflecting the relationship between agricultural production and the amount of water used, either from irrigation or precipitation. IWUE is defined in the literature by the following formula [19]:

(1) $$IWUE=\frac{Production\;(\;biomass\;or\;useable\;yield)}{Water\;used\;(irrigation+precipitation)\;}$$

In this work, we calculated IWUE based on the data obtained from the analyzed plot with wheat (*Triticum aestivum*) as the main crop, taking into account both irrigation and rainfall input. The data collected include a cultivated area of 1152 m^2^, a total water consumption of 1099.2 m^3^ (composed of irrigation and rainfall), and an average yield of 1.1 kg/m^2^. 

The IWUE of wheat (*Triticum aestivum*) varies depending on growing conditions, typically between 0.8 and 1.6 kg/m^3^. Factors such as climate, wheat type, and irrigation system influence IWUE. Climates with moderate temperatures and well-distributed rainfall contribute to more efficient water use, while fall wheat tends to have a higher IWUE than spring wheat due to better soil water use. Controlled irrigation and moderate deficit can also increase the IWUE.

The IWUE for the analyzed plot was calculated using the following formula:

(2) $$IWUE=\frac{1267.2\;kg}{1099.2\;m^{3}}\;=1.37kg/m^{3}$$

Interpretation of the results reveals that for each cubic meter of water used, 1.37 kg of wheat yield was obtained. Compared to the average values reported in the literature (0.8–1.5 kg/m^3^), this result reflects an efficient use of water resources in the cultivation process. The IWUE value of 1.37 kg/m^3^ is in the average range, indicating a satisfactory performance, but there is also potential for improvement. Compared to the optimal values of up to 2.0 kg/m^3^, this work suggests that with time, the learning capability of artificial intelligence can increase the IWUE index of our parcel to the optimal value.

### 4.3. Impact on Agricultural Sustainability

The proposed system significantly enhances agricultural sustainability by synergizing AI-driven irrigation optimization with low-cost IoT infrastructure, addressing three critical challenges: water scarcity, economic barriers, and ecological preservation. Through dynamic adjustments informed by real-time soil moisture and weather data (see Section 4.1), the system reduces water waste compared to static methods, alleviating pressure on dwindling hydrological resources in drought-prone regions [20]. Leveraging affordable components like ESP32 microcontrollers and sensors (see Section 3.1, Section 3.2, Section 3.3 and Section 3.4), it democratizes access to precision agriculture for smallholders [21]. By integrating AI and IoT technologies, the system enhances informed decision-making, achieving an irrigation water use efficiency (IWUE) of 1.37 kg/m^3^. This improvement not only safeguards local ecosystems but also advances sustainable and climate-resilient agricultural practices [22].

## 5. Discussion

The potential for using artificial intelligence (AI) to optimize irrigation processes is highlighted by the results of this study. The initial hypothesis is that AI can help reduce water consumption by adjusting the amount of irrigation to the actual needs of the crop and local climatic conditions. Although no large-scale experiments were conducted in this study, the results of the simulations indicate a very promising potential for integrating AI in agriculture to optimize water use efficiency and increase productivity.

In simple irrigation methods without AI support, farmers often rely on fixed routines or visual observations to decide when and how much water to use. Although this approach is less expensive, it has several limitations:

- Water waste: Frequent and chaotic irrigation leads to water run-off into already saturated soils, reducing irrigation water use efficiency (IWUE).
- Water stress: Without accurate measurements, crops can suffer from either overwatering or lack of water.
- Lack of adaptability: Traditional irrigation does not take into account how the weather or local soil varies.

In contrast, AI technologies prevent both waste and water stress by dynamically adjusting the amount and timing of irrigation. For example, an AI system can automatically reduce the amount of water applied before a rainstorm, which is not possible with manual systems that do not pay attention to the weather forecast.

Applying artificial intelligence to irrigation systems has major implications for agricultural sustainability. In the long term, this technology can help reduce the risks associated with climate change by using water resources more efficiently and increasing the resilience of agricultural systems. In addition, the use of AI could help small-scale farmers by reducing their operating costs and increasing their productivity.

A key aspect of this study is improving the irrigation water use efficiency (IWUE) index. AI algorithms enable precise irrigation. Over-irrigation and water loss are avoided. Simulations indicate a theoretical extrapolation of up to a 25% increase in IWUE by lowering water usage, but these estimates need to be validated by future experiments under real conditions.

There are several limitations that need to be addressed, although the results presented are promising. The main obstacles are a lack of field testing and a reliance on simulated data. The implementation of artificial intelligence in irrigation depends on several factors, including the existence of a traditional irrigation system. Without it, upfront costs can be significant, making technology adoption difficult. In addition, a poorly developed digital infrastructure in rural areas can make the process of implementing and developing smart solutions more difficult.

One of the main objectives in the development of the application should also be to take advantage of the sustainability of water resources in specific regional contexts. This approach could take into account how different regions are affected by drought and other climatic conditions, helping to personalize irrigation strategies and optimize water resource management based on the specific characteristics of the location. 

Optimizing the decision-making process is also an essential objective to ensure the continuity of the application and maintain the momentum of the decision-making process, otherwise the application will not be able to evolve with the rapid climate changes caused by global warming.

In addition, future research should focus on the development of scalable storage solutions for recorded data, capable of handling large amounts of information collected from multiple sensors. This could include the use of cloud platforms to ensure that data can be accessed quickly and analyzed in an efficient manner. 

Developing a learning and self-adapting system based on the results of the application’s past actions is another important aspect. By constantly analyzing and integrating the relationship between actions and their effects, the system can optimize the decision-making process and dynamically adapt to changing environmental and resource conditions. This self-adjusting mechanism would allow the application to learn from experience, reduce errors, and improve overall performance, ensuring that its full potential is realized. In this way, the application could evolve over time, becoming more and more efficient and offering more and more personalized solutions.

From a hardware point of view, there is a need for expansion of the sensor network. The exact number of sensors that need to be deployed depends on various factors, such as the wanted accuracy of the readings and the distribution of irrigation pipes. These sensors should include soil sensors to measure the pH value, air pressure, and wind sensors, as well as precipitation sensors to measure the amount and rate of rainfall. By monitoring climatic conditions more extensively and accurately, we can obtain more detailed data and make more informed decisions.

As potential future research directions, we plan to extend our work to provide data from our custom and advanced sensor systems, solar powered and fully integrated with our main application, expanding the information available to AI by measuring the slope, the custom feature that truly make every cultivable area unique, to better assess the micro-changes that could happen.

By analyzing the hardware feedback from our current system, we can see that the sensors chosen for the system might lack the desired precision; more sophisticated sensor solutions might appeal more to smaller parcels where the accuracy of irrigation might be critical, but for large-scale farms, the accuracy is less critical, and this system does not interfere with good irrigation decisions.

## 6. Conclusions

The originality of this study lies in its advanced integration of artificial intelligence (AI) with highly scalable nationwide IoT networks and interactive geospatial visualization, a paradigm shift from conventional IoT-based irrigation systems. Unlike existing solutions constrained by static thresholds, our framework employs dynamic AI models to analyze real-time microclimatic data, historical trends, and crop-specific requirements, enabling adaptive irrigation schedules responsive to erratic weather patterns—a critical innovation for drought-prone regions in Eastern Europe.

Key advancements include contextual AI recommendations powered by OpenAI’s API, which translates raw sensor data into actionable insights (e.g., “Delay irrigation during peak daylight hours to reduce evaporation losses by 30%”). The system also introduces national-scale risk mapping, capable of aggregating data from distributed IoT nodes to generate heatmaps that classify regions into risk tiers (high-risk, precautionary, optimal), enabling proactive resource allocation. While this study focuses on a single node, the system is designed for scalability across multiple nodes nationwide.

The system’s real-time monitoring of temperature, humidity, and soil moisture—combined with predictive analytics—provides a holistic view of national climate conditions. Scalability and adaptability are central to the framework: by integrating low-cost IoT sensors with OpenAI’s analytics, the system supports thousands of monitoring stations while remaining accessible to small-scale farmers. Interactive dashboards and customized reports bridge the gap between data collection and actionable strategies, offering crop-specific advice (e.g., “Increase maize irrigation during flowering by 20%”).

This paper provides a replicable model for global climate adaptation, with applications extending beyond agriculture, such as urban air quality monitoring. By democratizing AI-driven tools, the framework empowers stakeholders to address water scarcity, extreme weather, and food security challenges. In summary, this study advances precision agriculture by merging IoT scalability with AI-driven adaptability, offering a proactive, data-driven solution to enhance sustainability in vulnerable regions. Its integration of real-time analytics, geospatial risk mapping, and dynamic decision-making sets a new benchmark for climate-resilient agriculture.

## Figures and Tables

**Table 1:** Results based on different input parameters.

Question	Expected Response	Response Given by AI	Good (Yes/No)
Should we irrigate when soil moisture is 40%, temperature is 20 °C, humidity is 60%, and the crop is grain?	Yes	Yes	Yes
Should we irrigate when soil moisture is 45%, temperature is 18 °C, humidity is 70%, and the crop is sunflower?	No	No	Yes
Should we irrigate when soil moisture is 55%, temperature is 20 °C, humidity is 60%, and the crop is potato?	No	No	Yes
Should we irrigate when soil moisture is 50%, temperature is 22 °C, humidity is 55%, and the crop is oats?	No	Yes	No
Should we irrigate when soil moisture is 30%, temperature is 25 °C, humidity is 50%, and the crop is corn?	Yes	Yes	Yes
Should we irrigate when soil moisture is 60%, temperature is 28 °C, humidity is 80%, and the crop is rice?	No	No	Yes
Should we irrigate when soil moisture is 20%, temperature is 32 °C, humidity is 45%, and the crop is barley?	Yes	Yes	Yes
Should we irrigate when soil moisture is 35%, temperature is 35 °C, humidity is 30%, and the crop is soybean?	Yes	No	No
Should we irrigate when soil moisture is 40%, temperature is 24 °C, humidity is 60%, and the crop is peas?	Yes	Yes	Yes
Should we irrigate when soil moisture is 25%, temperature is 29 °C, humidity is 40%, and the crop is millet?	Yes	Yes	Yes

**Table 2:** Temperature, humidity and soil moisture data recorded.

Month	Temperature (°C)	Humidity (%)	Soil Moisture (%)
January	−3.8	85.0	68
February	−2.1	83.0	72
March	2.5	81.0	63
April	9.3	77.0	60
May	14.8	75.0	48
June	18.1	68.0	45
July	19.7	65.0	42
August	19.1	67.0	46
September	14.5	73.0	48
October	9.1	78.0	50
November	3.7	82.0	56
December	−1.5	86.0	59

## Data Availability

Data are contained within the article.
